# World AIDS Day — December 1, 2013

**Published:** 2013-11-29

World AIDS Day draws attention to the current status of the human immunodeficiency virus/acquired immunodeficiency syndrome (HIV/AIDS) epidemic worldwide. The theme for this year’s December 1 observance is “Shared Responsibility: Strengthening Results for an AIDS-Free Generation.”

The first cases of AIDS were reported more than 32 years ago in the June 5, 1981, issue of *MMWR*. Since then, an estimated 36 million persons worldwide have died from HIV/AIDS; an estimated 35.3 million persons continue to live with HIV infection (1).

In the United States, approximately 636,000 persons with AIDS diagnoses have died since the first cases were reported (2); an estimated 1.1 million persons continue to live with HIV infection (3).

Global efforts, including the U.S. President’s Emergency Plan for AIDS Relief (for which CDC is an implementing partner), provided antiretroviral therapy to approximately 9.7 million persons in low-income and middle-income countries in 2012, an increase of 1.6 million persons from 2011 (4).
